# Environmental DNA concentrations vary greatly across productive and degradative conditions, with implications for the precision of population estimates

**DOI:** 10.1038/s41598-024-66732-4

**Published:** 2024-07-29

**Authors:** Meghan B. Parsley, Erica J. Crespi, Tracy A. G. Rittenhouse, Jesse L. Brunner, Caren S. Goldberg

**Affiliations:** 1 School of the Environment, Washington State University, Pullman, WA USA; 2 School of Biological Sciences, Washington State University, Pullman, WA USA; 3 Department of Natural Resources and the Environment, University of Connecticut, Storrs, CT USA

**Keywords:** Molecular ecology, Conservation biology, Molecular ecology, Conservation biology

## Abstract

Population size is an important metric to inform the conservation and management of species. For aquatic species, environmental DNA (eDNA) concentration has been suggested for non-invasively estimating population size. However, many biotic and abiotic factors simultaneously influence the production and degradation of eDNA which can alter the relationship between population size and eDNA concentration. We investigated the influence of temperature, salinity, and ranavirus infection on eDNA concentrations using tadpole mesocosms. Using linear regression models, we tested the influence of each experimental treatment on eDNA concentrations at three time points before and during epidemics. Prior to infection, elevated temperatures lowered eDNA concentrations, indicating that degradation was the driving force influencing eDNA concentrations. During early epidemics, no treatments strongly influenced eDNA concentrations and in late epidemics, productive forces dominated as ranavirus intensity and dead organisms increased eDNA concentrations. Finally, population size was only an important predictor of eDNA concentration in late epidemics and we observed high levels of variation between samples of replicate mesocosms. We demonstrate the complexities of several interacting factors influencing productive and degradative forces, variation in influences on eDNA concentration over short time spans, and examine the limitations of estimating population sizes from eDNA with precision in semi-natural conditions.

## Introduction

Accurate estimation of species abundance is vital for conservation of endangered species, management of non-native invasive species, monitoring of commercially or culturally valuable species, and basic ecological study^1,2^. However, estimating population size is difficult in natural populations as it often requires large amounts of labor and time and can be invasive. Non-invasive sampling has major advantages over traditional monitoring techniques in time, cost, and impacts to individuals^3,4^. For instance, researchers can use capture-mark-recapture analytic methods with the “marks” being multi-locus genotypes generated from DNA collected from scat, hair, feathers, or other excrements^5^. Environmental DNA (eDNA), DNA shed by organisms into an environmental media such as water, snow, soil, or air^6–8^ also shows promise for non-invasively estimating population size.

Environmental DNA analyzed with species-specific or community metabarcoding approaches^9,10^ has been used to non-invasively detect species occurrence and determine the distribution of diverse taxa at local, regional, and broader scales^8,11,12^. Environmental DNA can also reveal information about populations^13^, such as spawning timing and locations^14^, estimating genetic diversity^15,16^, and, more relevant for this study, estimating species abundance^2,17^.

The simplest expectation is that concentration of a species’ eDNA in the environment increases with its abundance or density. Indeed, laboratory experiments in aquaria and mesocosms with populations of fish or amphibians often find strong correlations between the number of animals and eDNA quantities^2,18–21^. These laboratory studies suggest that estimates of population size of wild populations can be produced from concentrations of a species eDNA. However, translating these relationships to natural systems has been challenging. While some studies have found simple correlations between DNA concentrations and organismal abundance in natural systems^22^, or used a correction factor from known relationships^23^, in general the correlations tend to be much weaker than in the laboratory^2^, perhaps because there are productive and degradative influences in natural systems that obscure the relationship between eDNA and population size^24^. It may be possible to model important environmental variables, such as temperature^25^, but to do so requires a deeper understanding of the biotic and abiotic factors that influence the production and degradation of eDNA.

We know of many factors that influence rates of eDNA degradation in laboratory and natural conditions. Acidic conditions^26,27^, hotter temperatures^26,28,29^, UV radiation^26,30^, and presence of microorganisms and extracellular enzymes^31^ all increase decay rates of eDNA. Less is understood about the factors influencing eDNA production. Baseline or average production rates appear to vary among species^32–34^ and developmental stage (i.e., juveniles compared to adults;^35^). Environmental DNA shedding rates also increase with temperature^36–38^, food intake^39^, activity levels and energy expenditures^33^, and perhaps metabolic rate more broadly^40^. For instance, some studies have found that warmer temperatures strengthen the relationship between eDNA and abundance in lab^37^ and field conditions^25^, which suggests that increased eDNA production may be more important than rates of degradation at warmer conditions, or that higher degradation rates smooth out the relationships between eDNA concentrations and population sizes. Physiological stress, salinity, dissolved oxygen, and disease status may also influence eDNA production rates^40^, but these factors remain untested. For example, parasite infections are likely to change the production of host eDNA because of their wide-ranging effects on host physiology, including increasing metabolic rates and often eliciting a physiological stress response, as well as their damage to host tissues^41,42^, which may increase shedding of DNA-bearing materials from the host^43,44^.

**Figure 1:**
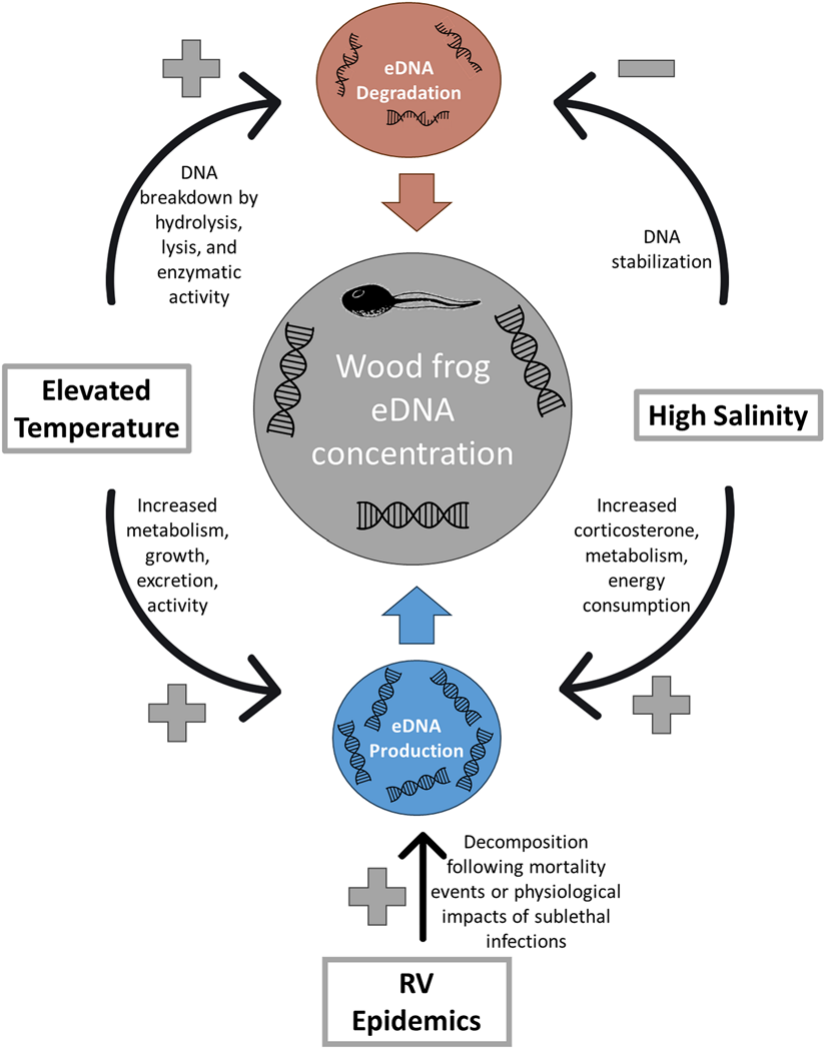
Influence diagram showing hypothesized impact (+ / − ) of each experimental treatment on eDNA production and degradation rates leading to wood frog eDNA concentrations collected from sampled mesocosms. RV = Ranavirus.

In natural systems, multiple biotic and abiotic factors are likely influencing production and degradation rates simultaneously, which can lead to a complex causal web leading to eDNA concentrations for a particular target population (Fig. 1). Experiments are needed to empirically determine how the multiple, potentially countervailing influences of realistic abiotic and biotic conditions on eDNA production and degradation balance and lead to population-level eDNA concentrations. Understanding these complex relationships is necessary to successfully use eDNA concentrations to estimate organismal abundance in natural systems.

**Figure 2:**
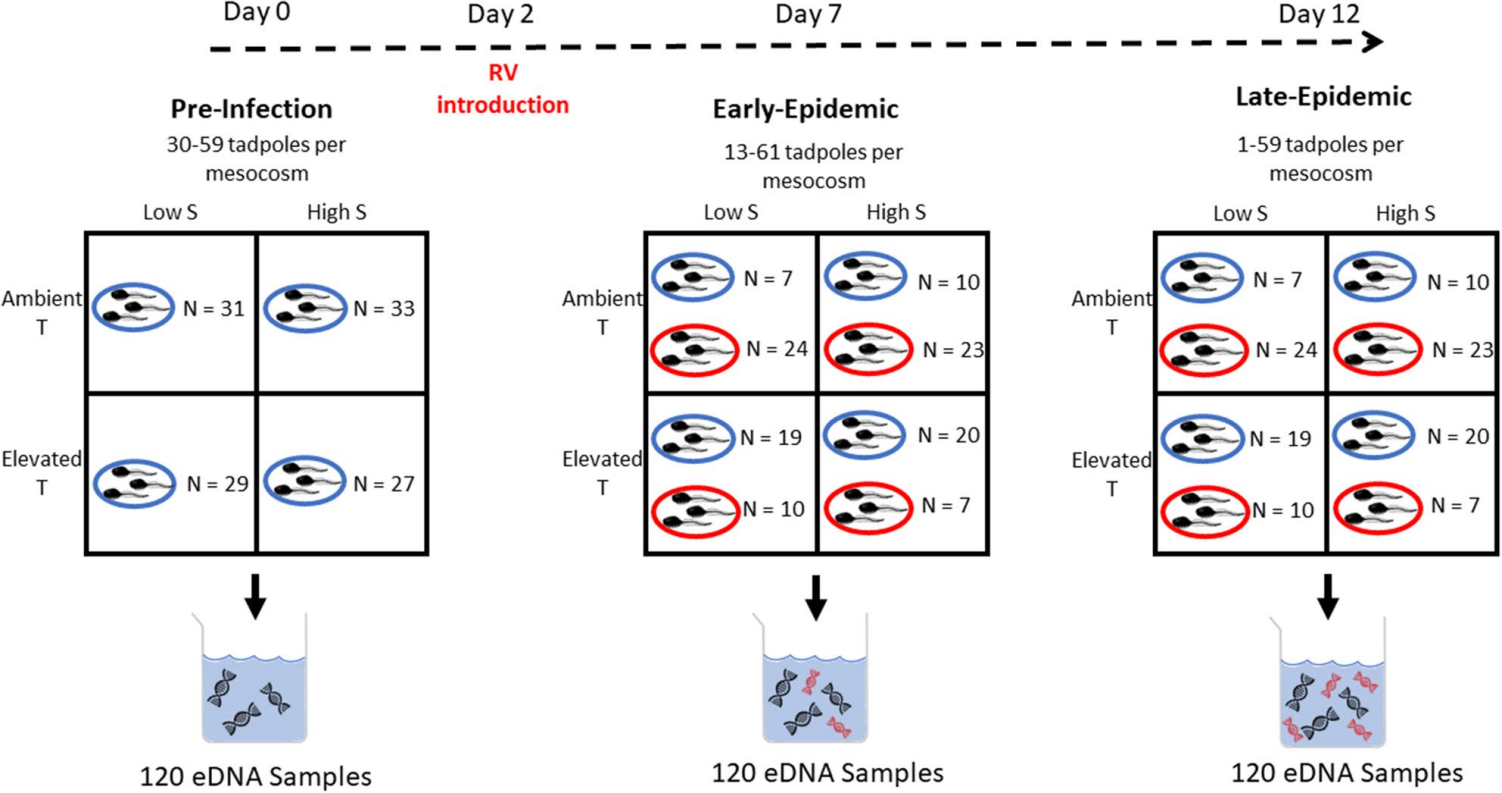
Representative diagram of experimental setup and timeline of the three environmental DNA sampling sessions (pre-infection, early-epidemic, and late-epidemic). Each sampling session involved the fully factorial experimental design of temperature (T) and salinity (S) treatments among the 120 replicate mesocosms (N). Ranavirus (RV) infected mesocosms are shown in red and control mesocosms are shown in blue with associated sample sizes for each treatment. In the pre-infection sampling session, all mesocosms in the associated temperature and salinity treatments were classified as controls. Environmental DNA samples were analyzed for wood frog eDNA (black) in all samples and ranavirus eDNA (red) after the introduction of ranavirus to the experimental mesocosms.

Here we tested the influence of three abiotic and biotic factors on the concentration of eDNA sampled from replicate mesocosm populations of larval wood frogs (*Lithobates sylvaticus*). We manipulated water temperature to simulate warming climate conditions (ambient and ~ 3 °C above ambient), salinity to mimic road salt runoff conditions (~ 0.113 and 1.718 mS/cm) and the occurrence of ranavirus (*Frog Virus 3, Ranavirus*, *Iridoviridae*). Ranavirus is a common pathogen in lentic systems^41^. that can infect many amphibian taxa, is transmitted through water and often impacts a large portion of the population and causing legions, necrosis, and hemorrhaging^41,42^. In addition, ranavirus susceptibility is influenced by external environmental factors and stressors^41,44^ and die-offs are common in wood frog populations^45^. We manipulated these experimental treatments in a factorial design to understand their individual and combined effect on concentrations of wood frog eDNA (Fig. 1). We used a high degree of replication, with ~ 30 nearly identical populations for each environmental treatment (temperature and salinity) and exposed a subset of each treatment group to ranavirus (Fig. 2). This provides a unique power to detect effects of the treatments with and without the presence of ranavirus throughout the entirety of the experiment. We collected eDNA samples from each mesocosm on three separate occasions to determine the influence of disease as an experimentally initiated epidemic progressed through the replicate experimental populations: prior to pathogen introduction (pre-infection), 5 days post-introduction (early-epidemic), and 10 days post-introduction (late-epidemic; Fig. 2). We analyzed each sampling session separately to better understand the influence of our abiotic environmental factors (temperature and salinity) as the epidemics developed. The relatively constant population sizes across mesocosms in our experimental design gave us the power to detect influence of our experimental factors on eDNA, which we focus on here across a relatively narrow range of population sizes.

## Results

**Figure 3:**
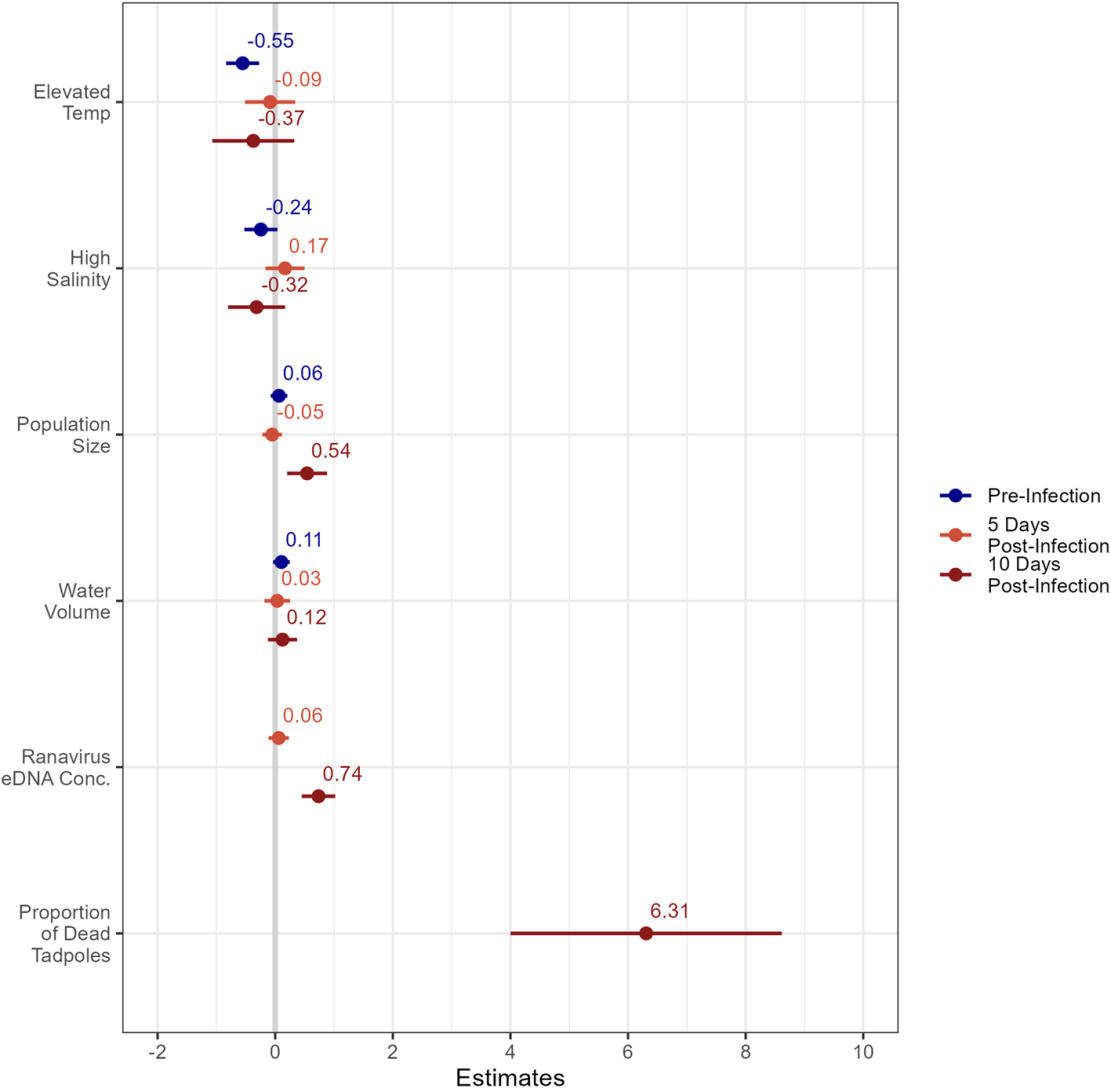
Forest plot of model parameter estimates and 95% confidence intervals for each of the three eDNA sampling sessions. Estimates for population size, water volume, and ranavirus eDNA concentration reflect scaled and centered data and have not been back transformed.

**Figure 4:**
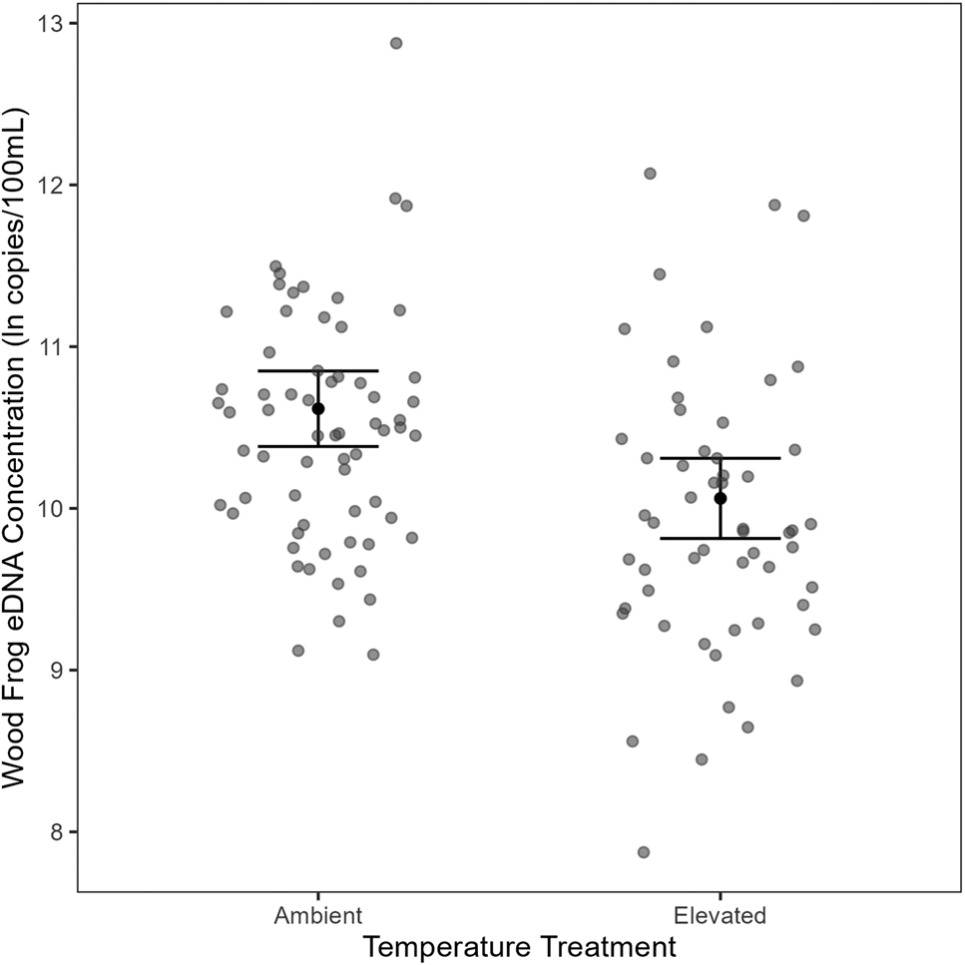
Pre-infection marginal effects plot of the influence of ambient and elevated temperature treatments on natural log transformed wood frog eDNA concentrations. Whiskers represent 95% confidence intervals and data points are shown as gray circles.

We modeled natural log transformed concentrations of wood frog eDNA (ln copies/100 mL) as a linear function of temperature treatment, salinity treatment, population size, and water volume in the mesocosm at the time of sampling to account for slight differences among mesocosms (mean 140 L, range 105–189 L) that could influence eDNA concentration. During the pre-infection sampling session, mesocosms with elevated temperatures had 42% (95% CI = 23.91–56.62%) lower wood frog eDNA concentrations than the ambient controls, after accounting for all other variables (Figs. 3 and 4). Neither salinity nor water volume had a notable association with wood frog eDNA concentrations (Fig. 3). Finally, there was no evidence that wood frog eDNA concentrations were associated with population size, though there was only a two-fold difference in numbers of tadpoles per mesocosm (from 30 to 59 tadpoles) and large variation among eDNA concentrations in each treatment (Figure S1). This linear model explained a small fraction of the variability in wood frog eDNA concentrations (*R*^2^_adj._ = 0.127).

At the early epidemic sampling timepoint, seven days later, we did not find evidence that any of the experimental variables associated with wood frog eDNA, including ranavirus eDNA concentrations (Fig. 3). Previous work has shown that ranavirus eDNA levels in natural wetlands correlate with infection intensity in individual tadpoles^46,47^. We thus expected that ranavirus eDNA concentrations would indicate the intensity of infections at the population level, which might lead to increased eDNA shedding by the hosts as cells are lysed, membranes become more permeable, and epidermal cells layers slough before tadpoles died. Yet ranavirus eDNA concentrations, which varied from 384 to ~ 300,000 copies/100 mL in ranavirus treatment mesocosms, did not predict a notable change in wood frog eDNA, at least at this sampling time. We did not detect an influence of temperature, salinity, or water volume on wood frog eDNA concentrations. Finally, population sizes varied over 4.5-fold, from 13 to 61 tadpoles per mesocosm due to tadpoles metamorphosing and sparse die-offs (metamorphosed and dead animals were removed daily leading up to this sampling) but there was no evidence that population size was associated with wood frog eDNA concentrations. Overall, our linear model explained little or no variation in wood frog eDNA concentrations (Fig. 3; *R*^2^_adj._ = − 0.018).

**Figure 5:**
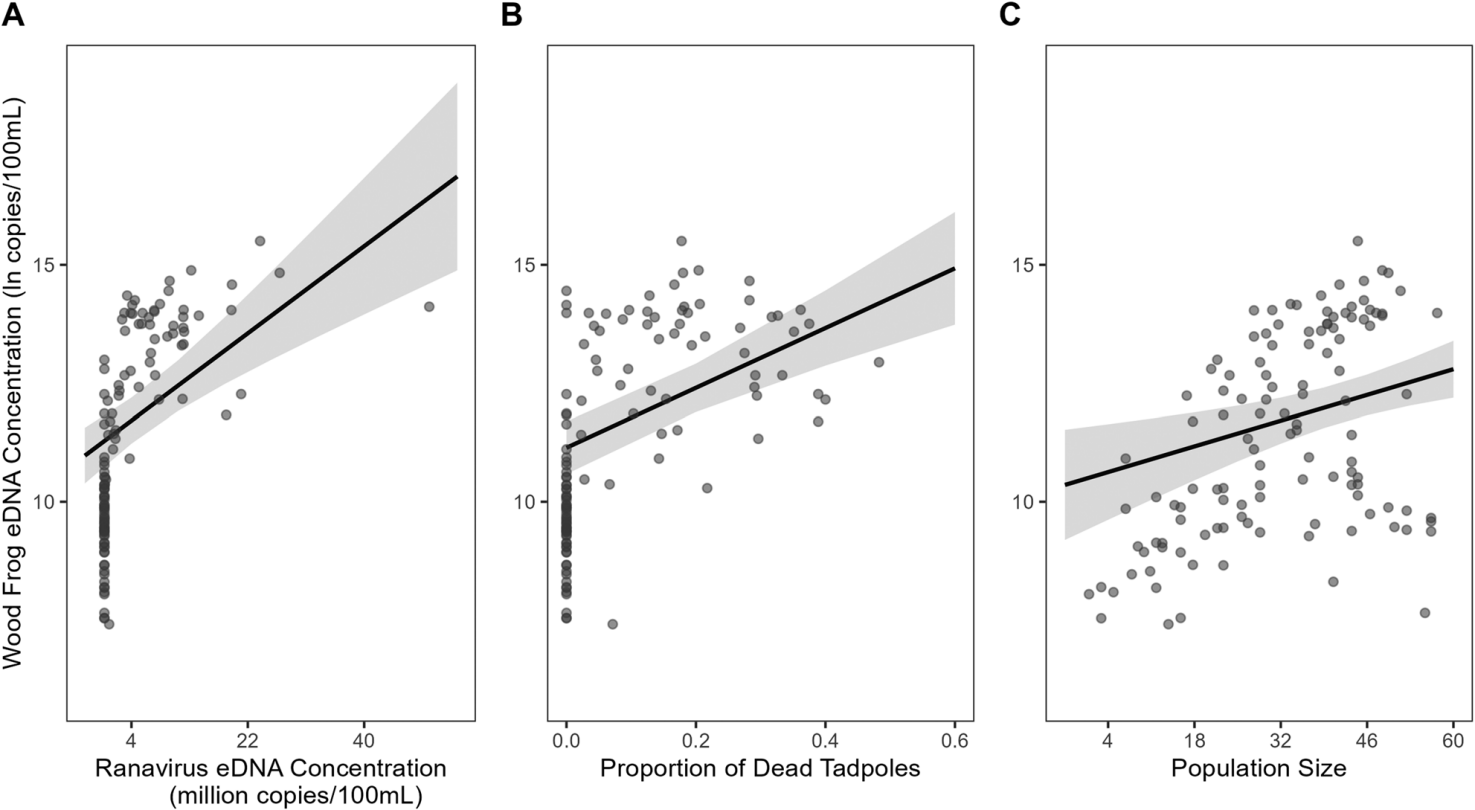
Marginal effects plots with data points for each of the three strongly influential factors on natural log transformed wood frog eDNA concentrations in the late-epidemic model: ranavirus eDNA concentration (**A**), proportion of dead tadpoles (**B**), and population size (**C**). Shaded areas represent 95% confidence intervals.

During the late epidemic sampling session (10 days post ranavirus introduction), epidemic mortality was clearly underway, meaning that large numbers of dead tadpoles were found and removed from each ranavirus mesocosm daily. Because eDNA samples were collected prior to daily censuses of mortality, our measure of population size includes tadpoles that died within the prior day, which may have been shedding more eDNA than live, intact tadpoles. We thus included in the model for this sampling session the proportion of dead tadpoles present in the mesocosm on the day of sample collection. We found that wood frog eDNA concentrations increased significantly with ranavirus eDNA concentration—an increase of ~ 7 × 10^6^ ranavirus eDNA copies/100 mL increased wood frog eDNA concentrations by 109% (95% CI = 57–178%; Fig. 5A)—as well as with the proportion of dead tadpoles—an increase in the proportion dead of 10% increased wood frog eDNA concentration by ~ 5000% (95% CI = 537–55,105%; Fig. 5B). Finally, we observed an increase in wood frog eDNA concentrations with population size, which ranged from 1 to 59 tadpoles per mesocosm. An increase of 10 tadpoles per mesocosm increased wood frog eDNA concentrations by 50.9% (95% CI = 15.9–100.2%; Fig. 5C). We did not detect an influence of salinity or temperature treatment on wood frog eDNA concentration at this sampling timepoint (Fig. 3) despite the presence of epidemic control mesocosms, but our model did explain a substantial fraction of the variation in the data (*R*^2^_adj._ = 0.614).

## Discussion

Our goal was to determine how two abiotic factors, elevated temperature and salinity, and a largely unexplored biotic factor, the occurrence of an epidemic of infectious disease, altered the concentration of population-level amphibian eDNA, presumably through their influences on rates of shedding or degradation (Fig. 1). While we found that elevated temperatures reduced wood frog eDNA concentrations (Fig. 4) and population-wide infection intensity and host mortality increased eDNA concentrations (Fig. 5), these results were only observed at certain points in time (pre-infection and late epidemic, respectively). Moreover, the influence of epidemic far exceeded that of wood frog tadpole population size. Collectively this suggests that inferring population size from eDNA concentrations of a target species may be exceedingly complex and simple adjustments for temperature or other factors may be insufficient to achieve even order-of-magnitude accuracy for smaller populations.

Environmental DNA concentrations were substantially reduced in the elevated temperature treatment relative to the ambient treatment in the pre-infection samples. Elevated temperatures are known to increase eDNA shedding rates^36^, especially in ectothermic aquatic organisms where metabolism, excretion, growth rates, and activity levels are likely to increase at elevated temperatures^40^. However, elevated temperatures can also increase cell and organelle lysis, hydrolysis and oxidation of DNA, and increased microbial activity that directly impact DNA integrity^47^, which increases rates of degradation of eDNA from fish^28,29,36,48^, amphibians^26^, and macro-invertebrates^49^. Our results suggest that increased degradation exceeded any increase in eDNA shedding, which has been observed in other systems^28,29^. However, prior work used more extreme temperature manipulation, from 5 to 15 °C between treatments. Our results suggest that even just 3 °C can have a marked impact on eDNA concentrations.

Importantly, we only found evidence that temperature influenced concentrations of wood frog eDNA in the earliest sampling period. In later samples of the same mesocosms, which were collected just days later, the estimated effects were smaller and less precise (Fig. 3). This might be due, in part, to the influence of temperature being swamped by those of the ranavirus epidemic in later sampling time points. However, uninfected control mesocosms were sampled throughout the study and in the early epidemic samples ranavirus-related parameters did not have any apparent influence on wood frog eDNA, which should have allowed for the effect of temperature to have been apparent in these samples. Alternatively, increasing overall ambient temperatures as the experiment progressed may have shifted into ranges above that where temperature effects are meaningful. Maximum water temperatures in ambient mesocosms stayed consistent, whereas maximum water temperatures for elevated temperature mesocosms increased by 4 °C over the course of the experiment. Despite this, the overall influence of temperature on wood frog eDNA concentrations appears inconsistent.

High salinity conditions can affect both production and degradation of eDNA. High salinity may cause stress to individuals and result in reduced activity, weight, and survival^50,51^, though we did not detect a difference in survivorship between salinity treatments. High salinity environments may also reduce rates of eDNA degradation^52^, although the evidence is weak, especially in marine systems^53,54^. In any case, we did not observe any influences of high salinity on eDNA concentrations for wood frogs.

The ranavirus epidemic had a very pronounced influence on wood frog eDNA concentrations, but only after tadpoles developed intense infections and began to die in large numbers. Early in the epidemic, five days after the virus was introduced into the mesocosms, ranavirus eDNA concentrations had essentially no predictive power, although the range of concentrations was relatively narrow (384 to ~ 300,000 copies/100 mL). Just five days later ranavirus eDNA concentrations were ~ 4,000,000 copies/100 mL on average (range 2.9 × 10^4^–4.9 × 10^7^), which is on the order of magnitude of ranavirus eDNA found during die-offs in natural ponds^46^, and many animals were dying. At this point wood frog eDNA concentrations increased by an order of magnitude on average for ranavirus infected mesocosms (from 32,000 in early-epidemics to 830,000 copies/100 mL in late-epidemics). Both ranavirus eDNA and the proportion of the population that died in the day prior to sampling were important predictors of wood frog eDNA concentrations. The high viral titers are likely indicative of moribund tadpoles in later stages of ranavirosis^46^, and who were thus likely hemorrhaging, experiencing necrosis of the liver and pro- and meso-nephros and even shedding their epithelial tissues^42^. That is, these intensely infected tadpoles were probably shedding much more DNA-bearing materials into the water than less intensely infected tadpoles five days earlier. Dead tadpoles were similarly losing their integrity and presumably shedding eDNA into the water, as has been seen in other natural systems^14,55^. Thus, we suspect that the strong influence of ranavirus epidemics on wood frog eDNA concentrations is largely attributable to a massive increase in eDNA production or shedding.

Tadpole population sizes were only strongly associated with wood frog eDNA concentrations at the last sampling time, late in the epidemic. To a degree, this likely reflects the narrower range of tadpole densities during the pre-infection (30–59 tadpoles per mesocosm) and early epidemic (13–61 tadpoles) samples, compared with the late epidemic samples (1–59 tadpoles). Prior studies that have found strong correlations between eDNA concentrations and population sizes in mesocosms had a similarly large or large range of population sizes: 1–121 in a study with bullfrog tadpoles^20^ and 1–64 with wood frog tadpoles^18^. However, given the large degree of replication and the detailed knowledge of the several confounding factors, both of which should have let us unmask otherwise hidden relationships, we were still unable to find a clear relationship over narrower ranges (e.g., across the early epidemic samples) in these small and controlled mesocosms. This suggests a limit to the precision of eDNA-based estimation of absolute population sizes and highlights an important disparity in the desired application to natural systems. While relative abundance estimates from eDNA concentrations are more achievable [e.g.,^37^], estimating absolute abundance/density is desired from eDNA concentrations^23,24^ and provides more tangible information to benefit biodiversity monitoring and conservation^56^. However, environments with natural populations are subject to much lower proportions of water sampled to the full amount of water in a natural system and numerous interacting environmental factors influencing production, degradation, and transport of eDNA compared to what is modeled in experimental mesocosms^2,24^. These added layers of complexity are likely to present many more challenges to estimating abundance from eDNA concentrations. Even more worrying, while we were able to measure all of the variables we expected would influence wood frog eDNA production and degradation, we were still unable to explain a substantial fraction of the variation in wood frog eDNA concentrations (*R*^2^_adj._ < 0.13), except in the last sampling time, during epidemic mortality (*R*^2^_adj._ = 0.626).

One explanation for our inability to explain or predict the variation in wood frog eDNA is the amount of sample-to-sample variation among replicate populations. In the pre-infection time period, where tadpole populations were most similar in population size and condition, we observed a range of eDNA concentrations that spanned one to two orders of magnitude difference among replicate mesocosms within a treatment (Figure S1). Such variability is to be expected from prior work, for instance, eDNA concentrations have been shown to vary tenfold among individual fish^17^ and 50-fold among individual amphibian larvae^26^. At a population level, replicates of 64 wood frog tadpoles housed in mesocosms varied over time by 100,000-fold copies/reaction^18^ and laboratory housed populations of 30 salmon had higher variability of eDNA concentrations compared to smaller population sizes of 5, 10, or 20 fish^19^. We assumed that our mesocosms were fairly well-mixed because they were small (190 L), exposed to natural wind conditions, contained UV filtration pumps that circulated water, and allowed natural swimming patterns of the tadpoles. However, it is important to note that we collected relatively small sample volumes (100 mL; 0.052% of the full volume) and did not include multiple sampling replicates per mesocosm. It is possible that increasing sample volume or collecting multiple replicate samples may be helpful in improving precision of eDNA quantification estimates by reducing the impact of potentially collecting large eDNA clumps^57,58^. However, this demonstrates that, even beyond the effects of biotic and abiotic factors, relating population sizes to eDNA concentrations with any precision may still be challenging.

Overall, we find that temperature and disease can strongly affect concentrations of a target species’ eDNA, but in complex ways that complicate the task of inferring population size from eDNA concentrations. While it is possible, in principle, to correct for the relationship between eDNA and abundance for the many interacting, often countervailing influence of biotic and abiotic factors that shape eDNA production and degradation rates, our results suggest these influences change over short periods of time and are highly context specific. Elevated temperatures reduced wood frog eDNA, presumably because rates of degradation increased more than production, but this was only apparent at the earliest sampling time. Ranavirus epidemics dramatically increased wood frog eDNA, but only when tadpoles were moribund or dying. Wood frog abundances were only strongly associated with their eDNA concentrations when abundance varied by two orders of magnitude, and this relationship was still small relative to the effect of the moribund and recently dead animals. And, as has been observed in prior studies, there was enormous variation in estimates of eDNA concentrations among seemingly very similar replicate populations. Thus, there are important limits to the precision with which eDNA concentrations can reliably estimate abundance and this relationship is likely to be more challenging in natural conditions where populations and habitats are larger and more complex and additional environmental factors are influencing eDNA concentrations. These limitations may be especially important in conservation contexts where population sizes are low and small changes can represent influential trends.

## Methods

### Mesocosm Setup

Our experimental setup consisted of 120 total mesocosms in an outdoor mesocosm facility at the University of Connecticut (Storrs, CT) with approximately 30 replicates of four environmental treatments (Fig. 2). The treatments included a full factorial design with two temperature treatments (ambient and elevated, ~ 3 °C above ambient) and two levels of salinity (low and high) randomly assigned to each mesocosm using a computer based random order generator. Mesocosms were 190 L oval tanks (Behlen Country, 214 Poly Round End Sheep Tank) filled with well water and inoculated with zooplankton and phytoplankton. Each mesocosm was equipped with a lid made of 50% PAK knit shade cloth (PAK Global, Cornelia, GA, USA), a temperature probe, 10 large, dry oak leaves as a natural substrate for tadpoles, substrate for algae growth for tadpole diets, and food source for plankton, and aquarium heaters (Submersible Heater GH300 from Aquatop) in elevated temperature treatment mesocosms. A UV filtration system (9 W from AA Aquarium) was also present in each mesocosm to ensure water clarity throughout the experiment and aid in tadpole observation. Since UV exposure has shown to increase eDNA degradation^26^, these filtration systems may affect eDNA degradation rates in our experiment. However, the increased degradation rate should be the same in every mesocosm, as a UV filter was present in each regardless of treatment, making the comparison among treatments valid. High salinity mesocosms were treated with road salt to mimic conditions in wetlands exposed to road salt runoff^59,60^ and averaged 1.718 mS/cm. Low salinity treatment mesocosms did not get any salt additions to the well water and averaged 0.113 mS/cm. We collected wood frog eggs from three natural populations in Mansfield, CT and stocked 60 tadpoles per mesocosm. Tadpoles were fed 5 g of ground alfalfa pellets per mesocosm as needed until virus exposure.

Immediately following the first eDNA sampling (described below), we censused the tadpoles in each mesocosm. Throughout the experiment, we monitored mesocosms daily to track the mortality or the metamorphosis of every tadpole in the mesocosm. We removed dead tadpoles and metamorphosing frogs when front legs developed, using a clean net for each individual and wearing single-use gloves when touching the water or animals. To estimate the population size in each mesocosm for the successive eDNA sampling sessions, we subtracted the number of deceased tadpoles or removed metamorphs between the initial census and the following sampling date. There was some variability in the accuracy of the original census as well as the detection of every dead individual throughout the experiment, which is likely due to the fast rate of decomposition of amphibian larvae in water^61^. Census errors resulted in more individuals being pulled out of the mesocosm over the course of the experiment than were counted on the census day (N = 22 mesocosms) and non-detections of deceased tadpoles resulting in fewer individuals being recovered over the course of the experiment than were counted on census day (N = 85 mesocosms). For mesocosms with census errors, we corrected the original count with the number of tadpoles that were removed from the mesocosm throughout the course of the experiment and recalculated the population sizes for the successive sampling session. For mesocosms with non-detection errors, we cannot distinguish between non-detection of deaths or a combination of census errors and non-detection of deaths. The average discrepancy between the number of tadpoles removed during the experiment and the number censused in the beginning was 4 tadpoles (range − 18 to 23 tadpoles). We distributed this error across the second and third eDNA sampling sessions by subtracting 2 individuals from each of the originally estimated population sizes. Population sizes averaged 47.41 tadpoles in the first eDNA sampling session (range 30–59), 45.97 tadpoles in the second sampling session (range 13–61), and 32.39 tadpoles in the third sampling session (range 1–58).

We introduced ranavirus-infected tadpoles or mock-infected tadpoles (controls) into each mesocosm when tadpoles reached an average Gosner stage of 36^62^. A single infected tadpole was introduced to 23 high salinity-ambient temperature and 24 low salinity-ambient temperature mesocosms and 7 high salinity-elevated temperature and 10 low salinity-elevated temperature mesocosms for a total of 64 ranavirus mesocosms. A single control mock-infected tadpole was introduced to 3 elevated temperature and 4 ambient temperature mesocosms of each salinity treatment. If the tadpoles in a mesocosm had moved past an average Gosner stage of 36, mostly in the case of elevated temperature mesocosms, we did not introduce an infected tadpole. Instead, we reclassified these as control mesocosms for their respective treatments (Fig. 2). Reclassification occurred for two mesocosms in each of the ambient temperature treatments, 20 high salinity-elevated temperature and 16 low salinity-elevated temperature mesocosms for a total of 56 control mesocosms. The ranavirus-infected and mock-infected tadpoles were raised separately in high or low salinity environments, matching the experimental mesocosms and tagged with a visible implanted elastomer (Northwest Marine Technology, Anacortes, Washington, USA) injected on dorsal side of their body so they could be distinguished from the naïve tadpoles in the mesocosms. Then, 24 h prior to their introduction these tadpoles were injected with *Frog virus 3* ranavirus in the intraperitoneal cavity or for the mock exposed controls, with saline. We held the treated tadpoles separately 24 h post-injection to ensure survival prior to adding a single treated or control tadpole to each mesocosm to subsequently spread the virus to others in the mesocosm population.

Nets and other equipment used throughout the experiment were soaked in 10% bleach solution for at least 2 min, rinsed with water, and dried prior to reuse on another individual. All animal care procedures were approved and performed in accordance with the University of Connecticut IACUC protocol number A17-006 and are reported in accordance with ARRIVE guidelines.

### eDNA sampling and quantification

We collected 100 mL water samples from each mesocosm at three time points during the experiment: 2–4 days prior to ranavirus introduction around Gosner stage 34^62^ (May 21–23, 2019), five days post-ranavirus introduction (May 28-June 1, 2019), and 10 days post-ranavirus introduction (June 2–6, 2019) for a total of 360 samples. We collected samples from the surface of the water at the center of each mesocosm using new 532 mL Whirl Pak bags (Nasco; Fort Atkinson, WI) and disposable gloves prior to any other activity at the mesocosms that day to avoid disturbing the water or tadpoles. We filtered the water through sterile 47 mm, 0.45 µm cellulose nitrate filters in single-use filter funnels (Sterlitech) manually using a hand pump immediately after collection from mesocosms and preserved each filter in 95% molecular grade ethanol. At the time of eDNA sample collection we recorded current water volume, estimated using water level deficit from the top of the mesocosm, and number of dead tadpoles in the mesocosm the day sampling occurred. We also collected control eDNA samples from 10 randomly selected mesocosms prior to tadpole addition, the water sources used to fill mesocosms and clean supplies throughout the experiment, and the alfalfa pellets used to feed tadpoles to ensure no wood frog or ranavirus eDNA was added before or during the experimental period. Additionally, we collected water samples from the temporary housing of the wood frog eggs collected for the experiment to ensure no ranavirus DNA was brought into the experimental mesocosms.

We extracted DNA from one half of each filter using the Qiashredder/DNeasy Blood and Tissue Kit method as described in Goldberg et al.^63^ in a designated low quality/quantity, restricted access eDNA lab separate from the amplification lab space to avoid contamination and following eDNA best practices^9^. We measured eDNA concentrations using species-specific quantitative PCR (qPCR) assays for wood frogs (Table S1) and ranavirus^64^. We ran samples in quadruplicate 15 µL reactions for 50 cycles and used standard curves created with synthetic DNA fragments (gBlocks; Integrated DNA Technologies, Coralville, IA) of the target region for each assay from 10^6^ to 10^1^ run in duplicate to quantify each sample. We included negative controls at extraction and qPCR steps to check for contamination and used an internal positive control in all wells (IC; Qiagen, Hilden, Germany) to test for inhibition in each qPCR reaction.

Quantification of copy number was highly accurate, with R^2^ of the standard curves ranging from 0.99 to 1.00 for wood frog and 0.98–1.00 for ranavirus. Quantitative PCR efficiencies ranged from 92.5 to 101.6% for the wood frog assay and 88.9–98.7% for the ranavirus assay. The limits of detection (LOD), defined as the lowest standard concentration of template DNA that produced at least 95% positive replicates^65^, were 3 copies and 5 copies per reaction for the wood frog and ranavirus assays respectively. The limit of quantification (LOQ) determined by the discrete cutoff threshold of the lowest standard concentration that could be quantified with a CV value below 35%^65^ was 10 copies per reaction for both assays. We found no contaminating eDNA of wood frog or ranavirus in the mesocosms prior to introduction of tadpoles and did not detect ranavirus eDNA in the eggs that were used for stocking mesocosms for the experiment. Additionally, we did not detect ranavirus or wood frog eDNA in the alfalfa pellets used to feed tadpoles or water used to fill the mesocosms and clean supplies throughout the experiment.

Four extraction negatives tested positive for ranavirus DNA and two extraction negatives tested positive for wood frog DNA in two to four qPCR replicates indicating some level of contamination between samples during laboratory extraction procedures. All extraction batches where extraction negatives detected contamination corresponded to samples taken in the third sampling session when eDNA values were uncharacteristically high of those typical for environmental samples (some > 100,000 copies per qPCR reaction for ranavirus eDNA) and possibly influenced 73 out of 360 total samples across experimental treatments in the dataset. For ranavirus, contamination levels ranged between 5 copies and < 1 copies of DNA per reaction while wood frog contamination levels ranged between 2 copies and < 1 copies of DNA per reaction. We determined that due to the contamination levels being at or less than the LOD for each respective assay and orders of magnitude less than the eDNA amounts per sample, no correction for lab contamination was necessary. We pruned a total of six qPCR replicates from our dataset due to inhibition during the qPCR reaction. For the wood frog assay, we removed one qPCR replicate from each of three samples and for the ranavirus assay we removed one qPCR replicate from each of three samples. These pruned replicates occurred in different samples for each assay and may have been due to manual pipetting errors.

### Data analysis

We scaled wood frog eDNA concentrations from copies per qPCR reaction up to copies/100 mL of sampled mesocosm water and natural log transformed these values for linear regression modeling. We assessed how the different experimental treatments influenced eDNA concentrations among replicate populations of wood frog tadpoles as described in the main text, including temperature treatment, salinity treatment, population size, and water volume in models for each sampling session. We included ranavirus eDNA concentrations in early and late epidemic models as a measure of epidemic intensity in the populations as well as the proportion of dead tadpoles in the late epidemic model to account for die-offs. Prior to modeling, we scaled and centered continuous variables (population size, water volume, and ranavirus eDNA concentrations) and checked all predictor variables for collinearity using Pearson product correlations and multicollinearity using variable inflation factor (VIF;^66^). We did not find significant correlations (r < 0.7) or multicollinear variables (VIF < 3) between continuous variables for any of the three modeling datasets. Model assumptions were verified by plotting residuals versus fitted values and versus each covariate in the model and model validation indicated no problems with residuals in any of the three sampling session models. We calculated and plotted marginal effects for influential variables in each model using the packages *sjPlot* (version 2.8.10;^67^) or *ggeffects* (version 1.1.1,^68^). All analyses were completed using R version 4.1.0^69^.

### Supplementary Information

Supplementary Information.

Supplementary Dataset.

## Data Availability

All data generated and analyzed in this study are included in the Supplementary Dataset.
