# Supplementary material for: Environmental DNA concentrations vary greatly across productive and degradative conditions, with implications for the precision of population estimates
Source: Sci Rep. 2024 Jul 29;14:17392. doi: 10.1038/s41598-024-66732-4 (PMC11286860; doi:10.1038/s41598-024-66732-4)
Supplement: Supplementary file 1 — Supplementary Information. [file 41598_2024_66732_MOESM1_ESM.pdf]

Supplemental Information For:

Environmental DNA concentrations vary greatly across productive and degradative conditions, with implications for the precision of population estimates

Meghan B. Parsley*[1], Erica J. Crespi[2], Tracy A.G. Rittenhouse[3], Jesse L. Brunner[2], Caren S. Goldberg[1]

[1]School of the Environment, Washington State University, Pullman, WA, USA

[2]School of Biological Sciences, Washington State University, Pullman, WA, USA

[3]Department of Natural Resources and the Environment, University of Connecticut, Storrs, CT, USA

*Corresponding Author: Meghan B. Parsley; Meghan.parsley@wsu.edu

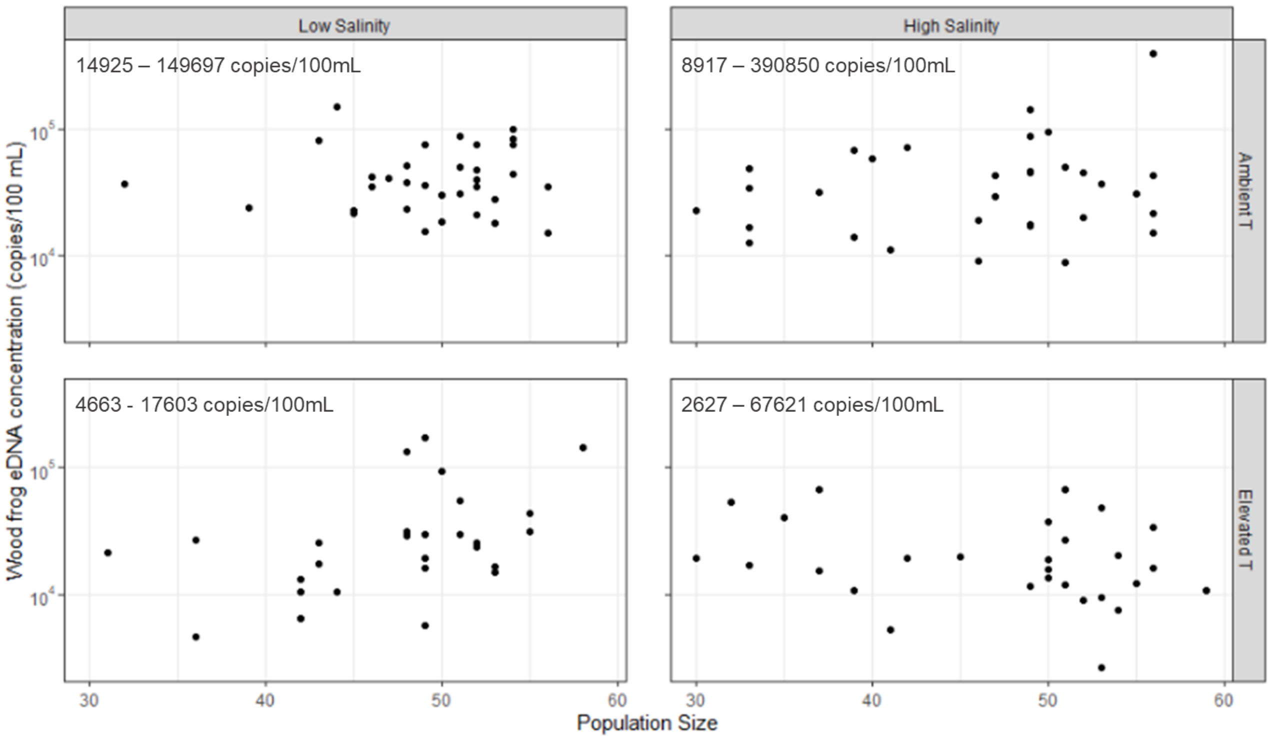

Figure S1. Scatter plots of eDNA concentrations and population sizes for each replicate mesocosm in the four environmental treatments prior to the exposure to ranavirus. Ranges of values for that treatment group are listed in the top left corner of each plot.

Table S1. Quantitative PCR assay design for wood frog (*Lithobates sylvaticus*) used in this study. Samples analyzed in 15 µL reactions using 1X TaqMan Environmental Master Mix (Thermo Fisher Scientific, Waltham, MA), 0.2 µM of each primer and probe, IC Assay and DNA (QuantiFast Pathogen PCR + IC Kit, Qiagen, Hilden, Germany), and 3 µL of sample. Cycling began with 10 minutes at 95°C followed by 50 cycles of 95°C for 15 seconds and 60°C for 60 seconds. This assay was validated against tissue samples of Cope's gray treefrog (*Hyla chrysoscelis*), spring peeper (*Pseudacris crucifer*), American bullfrog (*Lithobates catesbeianus*), green frog (*Lithobates clamitans*), and northern leopard frog (*Lithobates pipiens*) for specificity per Goldberg et al. (2016). Sequences used for assay design are from Lee-Yaw et al. 2008.

Primer/Probe	Sequence
LISY-F	5'-MCC TCA CCC GAT TTT TTA CRT-3'
LISY-R	5'-TGA TGA AGG AAG AGA AGG TGG ATT-3'
LISY-Probe	Cy5-TTC A+TT +ATC GC+A GC+T GC+A AGT AT-BHQ_2[*]

[*]Bases with + before denote locked nucleic acid (LNA) bases present in the probe

Lee-Yaw JA, Irwin JT, Green DM (2008) Postglacial range expansion from northern refugia by the wood frog, *Rana sylvatica*. Molecular Ecology, 17(3):867-884.
